# Effects of Humic Substances on the Growth of *Pseudomonas plecoglossicida* 2,4-D and Wheat Plants Inoculated with This Strain

**DOI:** 10.3390/microorganisms10051066

**Published:** 2022-05-22

**Authors:** Arina Feoktistova, Margarita Bakaeva, Maxim Timergalin, Darya Chetverikova, Aliya Kendjieva, Timur Rameev, Gaisar Hkudaygulov, Aleksey Nazarov, Guzel Kudoyarova, Sergey Chetverikov

**Affiliations:** 1 Ufa Institute of Biology, Ufa Federal Research Centre, RAS, Prospekt Oktyabrya 69, 450054 Ufa, Russia; feoktistova.arisha@yandex.ru (A.F.); margo22@yandex.ru (M.B.); belka-strelka8031@yandex.ru (D.C.); aliya_kendzieva@mail.ru (A.K.); rameevt@mail.ru (T.R.); bio.logos@yandex.com (G.H.); guzel@anrb.ru (G.K.); che-kov@mail.ru (S.C.); 2 Department of Environment and Rational Use of Natural Resources, Faculty of Business Ecosystem and Creative Technologies, Ufa State Petroleum Technological University, ul. Kosmonavtov 1, 450064 Ufa, Russia; nazarovam1501@gmail.com

**Keywords:** rhizosphere bacteria, *Pseudomonas plecoglossicida* 2,4-D, humic substances, humic and fulvic acids, wheat plants

## Abstract

Both rhizosphere bacteria and humic substances (HSs) can promote plant growth when applied individually and even greater effects of their combination have been demonstrated. We aimed to elucidate the relative importance of the stimulating effects of HSs on bacterial growth and the effects of the combination of bacteria and HSs on plants themselves. The effects of humic (HA) and fulvic acids (FA) (components of humic substances) on the growth of *Pseudomonas plecoglossicida* 2,4-D in vitro were studied. We also studied the effects of this bacterial strain and HSs applied individually or in combination on the growth of wheat plants. Although the 2,4-D strain showed low ability to use HSs as the sole source of nutrition, the bacterial growth rate was increased by FA and HA, when other nutrients were available. HSs increased root colonization with bacteria, the effect being greater in the case of HA. The effects on roots and shoots increased when bacteria were associated with HSs. FA+ 2,4-D was more effective in stimulating shoot growth, while HA + 2,4-D was in the case of root growth. The latter effect is likely to be beneficial under edaphic stresses.

## 1. Introduction

Plant growth-promoting bacteria (PGPB) [1,2,3] and humic substances (HSs, products of degradation of organic matter extracted from brown coal, peat, and other sources) [4,5,6] studied separately have been frequently shown to simulate plant growth and increase their crop yield. The interest in either PGPR or HSs continues unabated with numerous publications demonstrating their capacity to stimulate plant growth and increase their productivity (see ref. [7]). For example, recent publications demonstrated that PRPR from spp of *Bacillus* and *Trichoderma* can improve germination, seedling growth, and potassium uptake of soybean [8], *Azospirillum lipoferum* enhanced the flood-induced increase in lateral root growth of maize cultivars [9], *Bacillus cereus* mitigated heat stress in *Solanum lycopersicum* [10], and *Rhizobium leguminosarum* and *Paenibacillus polymyxa* increased the productivity of wheat under saline soil conditions [11]. Data on the effects of humic acids on plant growth and productivity are less abundant, but still frequently reported. Application of potassium humate by seed dressing and through soil application improved the soil properties, productivity, and fiber quality traits of cotton [12], humic acids enhanced cucumber shoot growth by up-regulation of genes encoding aquaporins, thereby increasing hydraulic conductance of their roots [13], and HSs enhanced root elongation, lateral root emergence, and plasma membrane H^+^-ATPase activity in maize roots [14]. Numerous articles promote the use of either PRPG or humates in agriculture as biostimulants. A recent review provided an analysis of a few reports on the prospect of HSs in combination with PGPB as “an alternative for sustainable agriculture” [15]. However, another review [16] emphasized that the numbers of scientific reports considering the action of PGPB in combination with HSs is extremely low in comparison with the great potential of these types of biological inputs. The lack of information on the topic leads to the fact that some important aspects remain unresolved. It has been shown that HSs can stimulate bacterial growth [17], but it remains unclear whether this effect is more important for stimulating plant growth than the direct effect of HSs on the plants themselves. Furthermore, HS is a mixture of HA and FA which differ in size, structure [18], and activity toward bacteria [19] and plants [4]. Therefore, it is of interest to compare the effects of HA and FA on the growth of bacteria and bacteria-inoculated plants, which, to the best of our knowledge, have never been investigated in the same experiment. Taking into account all of the above, the aim of the present research was to study the effects of HA and FA on the growth of *Pseudomonas plecoglossicida* and bacteria-inoculated wheat plants. The choice of bacteria and plant species was based on our previous experience with the effects of *Pseudomonas* ssp. on plants including wheat [20,21]. Particular attention was paid to root development due to the known effects of PGPR [9] and HSs [14] on root branching when applied separately, which is likely to be important for root colonization by bacteria.

## 2. Materials and Methods

### 2.1. Extraction of Humic and Fulvic Acids and Their Spectral Characteristics

The source of humic substances was brown coal from the Tyulganskoe deposit in the Orenburg region of the Russian Federation. Coal was mixed with 0.1 M KOH in a ratio of 1:10 and was extracted during the day. The precipitate was removed by centrifugation at 12,000 rpm for 10 min. Then, 0.1 M HCl was added dropwise to the supernatant until pH 3 was reached and stirred for 1 min. Fractions of FA (supernatant) and HA (precipitate) were separated by centrifugation at 12,000 rpm for 10 min, and the precipitate was washed once with cold distilled water. Samples of humic and fulvic acids were dried at 60 °C and weighed. Dried HS samples were stored until they were used to study their effects on plants and microbes. Our own studies and literature data show stability of these substances.

Humic substances (HA and FA) isolated from brown coal were analyzed by ^1^H NMR, ^13^C NMR, IR, UV spectroscopy, and UV spectrofluorimetry. ^1^H and ^13^C nuclear magnetic resonance (NMR) spectra were recorded on a BrukerAvanceIII pulse (Bruker, Germany) spectrometer with an operating frequency of 500.13 MHz (^1^H) and 125.47 MHz (^13^C) relative to the signal of the internal standard tetramethylsilane (TMS), D_2_O, 0.1 M NaOH. The IR spectra of the samples were recorded on an IRPrestige-21 spectrophotometer (Shimadzu, Japan) in a thin layer. UV spectroscopy was carried out on a UV-2600 spectrometer (Shimadzu, Japan) using solvent distilled water (0.05N NaOH). Fluorescence spectra were recorded on the spectrofluorimeter RF-600 (Shimadzu, Japan), λex = 310 nm.

### 2.2. Bacterial Strain and Culture Media

*Pseudomonas plecoglossicida* strain 2,4-D described in the article by Chetverikov et al. [22] and capable of accumulating indole-3-acetic acid (IAA) in culture media [23] was used in the present study. To study the effect of HA and FA isolated from coal and their joint extract, *P. plecoglossicida* 2,4-D bacteria were cultivated for 4 days in Erlenmeyer flasks on a thermostatically controlled shaker (160 rotation per minute) at 28 °C in the King B nutrient medium (g L^−1^): peptone, 20.0; glycerol, 10.0; K_2_HPO_4_, 1.5; and MgSO_4_·7H_2_O, 1.5, to which sterile humic additives were added before cultivation to yield a final concentration of 1 g L^−1^. HSs were sterilized by passing through a CHROMAFIL^®^Xtra PA-45/25 (Macherey-Nagel, Germany) syringe nozzle. A bacterial culture grown in King B medium without additional additives served as a control. The ability of *P. plecoglossicida* 2,4-D bacteria to use HSs as the sole source of carbon was also tested by adding HSs (1 g L^−1^) to Raymond media (g L^−1^): NH_4_NO_3_, 2.0; MgSO_4_·7H_2_O, 0.2; KH_2_PO_4_, 2.0; Na_2_HPO_4_, 3; CaCl_2_·6H_2_O, 0.01; and Na_2_CO_3_, 0.1, pH 7. To clarify the role of HSs in the nutrition of bacteria, they were added as a source of nitrogen to the Ashby medium (g L^−1^): K_2_HPO_4_, 0.2; MgSO_4_, 0.2; NaCl, 0.2; Na_2_MoO_4_, 0.006; CaCO_3_, 5.0; and sucrose, 20. The bacteria were precipitated from the King B medium by centrifugation (10,000 rpm) for 5 min, washed once, re-suspended in sterile water, and added to the Ashby or Raymond medium in an amount of 10 µL per 100 mL of medium.

Plant treatment bacteria were cultured for 3 days in liquid King B nutrient medium as described above for this medium. The number of cells in the cultures was measured by applying serial dilutions to King B medium with agar-agar (15 g L^−1^) and then counting the number of colony-forming units (CFU). The bacterial culture was diluted with sterile water to obtain a solution for spraying plants containing (1 ± 0.5) × 10^8^ CFU mL^−1^. To determine the IAA content in plants, the shoots and roots were homogenized and extracted with 80% ethanol. Alcohol extract was evaporated to an aqueous residue, centrifuged, and the aliquots of the supernatant were taken for analysis. Auxin concentrations in culture media and plants were immunoassayed as described previously [23].

### 2.3. Estimation of Root Colonization

Root colonization by bacteria was examined using a rifampicin-resistant version of *P. plecoglossicida* 2,4-D^rif^. The plant roots were carefully removed from the pots, the sand shaken off, placed in vials with sterile water, and shaken for 1 h. After that, the washings from the roots were cultured in nutrient agar medium (g L^−1^): NaCl, 5.0; peptone, 10.0; glucose, 2.0; yeast extract, 1.0; and agar, 15.0 to assess the total number of bacteria and in King B medium with the addition of 50 mg L^−1^ rifampicin as a selective agent for the isolation of bacteria *P. plecoglossicida* 2,4-D^rif^.

### 2.4. Experiments with Plants

Experiments were performed with bread spring wheat (*Triticum aestivum* L., cv. Kinelskaya). The plants were grown at 14 h photoperiod, day/night temperature regimes of 23–25/18 °C and irradiance of 400 µmol m^−2^ s^−1^ from Osram fluora L36/W77 (Munich, Germany). Wheat seeds were sterilized in 2% sodium hypochlorite solution for 10 min, then the seeds were repeatedly washed with distilled water and germinated for 3 days. Then, they were planted in pots with sand. Sand was used due to the absence of HSs in it. It also allowed easy separation of roots from the substrate and sterilization by calcinations to prevent the introduction of undesirable bacteria. The humidity of the sand was maintained at the level of 60% of the total moisture capacity. The treatment of plants was carried out on the next day. Additionally, 1 mL of 0.1% aqueous solution of HA or FA and/or suspension of bacteria *P. plecoglossicida* 2,4-D (10^8^ CFU mL^−1^) grown in King B liquid nutrient medium was sprayed on the soil and directly on the plants.

Growth parameters (weight and length of shoots and roots and number of lateral roots) were evaluated 7 days after plant treatment. Preliminary experiments have shown that this duration of plant growth is sufficient for the manifestation of the effects of both PGPR and HSs. With a longer cultivation of plants, a plexus of roots occurred, which made it impossible to separate the roots of each plant and calculate their laterals.

Data are expressed as means ± S.E., which were calculated in all treatments using MS Excel. Significant differences between means were analyzed by one-way analysis of variance (ANOVA) and (Duncan’s) test to discriminate means. The data were processed using Statistica version 10 software (Statsoft, Moscow, Russia).

## 3. Results

### 3.1. Spectral Characteristics of Humic and Fulvic Fractions

In the ^13^C NMR spectrum of HSs (solvent 0.1M NaOD in D_2_O), signals were observed in the range (ppm): 165–195 (C=O—carbon of keto groups); 140–165 (aromatic carbon associated with O (CSp^2^-O)); 100–140 (aromatic carbon bound to aliphatic carbon (CSp^2^-CH)); 50–100 (aliphatic carbon bound to O (CSp^3^-O)); and 0–50 (aliphatic carbon associated with CH (CSp^3^-CH). The ^13^C NMR signals of both humates and fulvates were in these regions of the spectrum and correspond to the data of Kholodov et al. [24].

In the ^1^H NMR spectra, peaks were found in the range of 0.5–1.95 (C, H substituted aliphatic fragments), 1.95–3.1 (aliphatic fragments in position α to an electronegative group or aromatic ring), 4.7–6.0 (aliphatic fragments doubly substituted with heteroatoms), and 6–10 (CH-substituted aromatic fragments). The ^1^H NMR signals of both humates and fulvates were in these regions of the spectrum and correspond to the data [25].

In the IR spectra of HS, intense absorption bands at 2450–3350 cm^−1^ (HA-3363, total coal extract (E)-3400, FA-3417) were assigned to hydroxyl groups (phenolic, alcohol, and OH groups in carboxyl groups), 2910–2930 (HA-2917, E-2927) and 1370–1380 (HA-1377, E-1377, FA-1370) cm^−1^—referred to long methylene chains, and 2850–2870 (HA-2870, E-2852, FA-2850) cm^−1^—to the methyl end groups. Absorption bands at wavelengths of 1705–1720 (HA-1705, FA-1720) cm^−1^ corresponded to carboxyl groups (C=O for carboxyl groups), 1600–1650 (HA-1614, FA-1639) cm^−1^ indicated the presence of benzoid structures (C=C in aromatic systems), 1225 (HA-1265, E-1161, FA-1149) cm^−1^—OH groups in carboxyl groups, and 1030–1060 (H-1033, F-1060) cm^−^—OH groups of carbohydrates. 1650 cm^−1^ indicated C=N in imino groups, where H, F, and E are fractions of humic, fulvic, and intermediate fractions, respectively. These signals for either H or F are in accordance with the literature data [26,27].

For UV–visible spectra of sodium humates (0.08% solution of humic acid in 0.05 N NaOH): E^465^ = 3.42; E^665^ = 0.789. For 0.001% solution of humates: E^465^ = 0.042; E^665^ = 0.0097. E^465^/E^665^ = 4.33, which corresponds to the literature data [27,28]. For UV–visible spectra of sodium fulvates (0.10% solution of fulvic acids in 0.05 N NaOH): E^465^ = 0.109; E^665^ = 0.096. For a 0.001% solution of fulvates: E^465^ = 0.0011; E^665^ = 0.00036. E^465^/E^665^ = 3.3.

Fluorescence spectra of humates: λex = 310 nm (0.081% in 0.05% NaOH) and λmax (fluorescence) = 418 nm; of fulvate: λex = 310 nm (0.10% in 0.05 NaOH) and λmax (fluorescence) = 421 nm, which corresponds to [29] for either HA or FA.

### 3.2. Effects of Humic Substances on Proliferation of Bacteria In Vitro

A high rate of proliferation of bacteria (cell reproduction) was detected in the studied bacterial cultures. None of the tested humic substrates slowed down the growth of *P. plecoglossicida* 2,4-D (Table 1). On the contrary, at certain time intervals, a significantly higher titer of bacterial cells in the culture medium was recorded. For example, this was observed after 8 h from the start of cultivation during the first half of the exponential growth phase. The second period of favorable influence of humic substrates was observed after 48 h of bacterial cultivation after the complete transition of the culture to the stationary phase of growth, which made it possible to obtain a culture liquid ready for further use with a higher content of cells. It should be noted that HA and FA separated from the extract had a greater stimulating effect than the crude extract.

The ability of *P. plecoglossicida* 2,4-D to use humic substances as the sole source of nitrogen or carbon was also tested. On the first day of cultivation in Ashby’s (Appendix A Table A1) or Raymond’s media (Table 2), bacteria multiplied, possibly due to a small amount of nutrients introduced into the new medium in the form of dead cells, molecules absorbed by bacterial cells, or intracellular spare substances. Then, the reproduction of bacteria stopped. The final titer of their cells in a nitrogen-free Ashby’s medium was about 10^6^ CFU mL^−1^. Since the addition of HA, FA, or HSs did not enhance cell multiplication compared to the control, humus substances were not likely to be a significant source of nutrition for bacteria.

A final titer of bacterial cells obtained in Raymond’s medium with added FA was not different from the control. However, with HA, it was higher than in the control, which may indicate the use of humic acids as a source of nutrients, although the level of its usage is likely to be rather low.

Auxin concentration in bacterial culture media was not increased by HSs and was about 850 ng mL^−1^.

### 3.3. Root Colonization by Bacteria

Bacteria growing on nutrient agar can serve as an indicator of the total number of microorganisms in the studied samples. The number of these bacteria in root washing varied significantly between plants subjected to different types of treatment (Table 3). The introduced strain *P. plecoglossicida* 2,4-D^rif^ made a significant contribution to the increase in the number of bacteria. Its selective detection on the medium with rifampicin showed that the number of CFU of *P. plecoglossicida* 2,4-D^rif^ was comparable to the total number of bacteria in the same samples. On nutrient agar, the number of indigenous microorganisms, which possibly migrated from seeds, was several times lower than the introduced ones (data not shown). Thus, during first week after the introduction of *P. plecoglossicida*, 2,4-D^rif^ was the dominant species in the rhizosphere of wheat plants. Clearly, its effect on plants prevailed over the influence of indigenous microbiota. The results obtained indicate that humic acids influenced the development of bacteria in the wheat rhizosphere. In the presence of HA, the total number of bacteria in the rhizosphere of one plant increased by 2.6 times both in the variants without the introduction of *P. plecoglossicida* 2,4-D^rif^ and in the variants with its introduction. The stimulating effect of FA on bacterial growth was weakly expressed.

### 3.4. Treatment Effects on Plants

**Figure 1:**
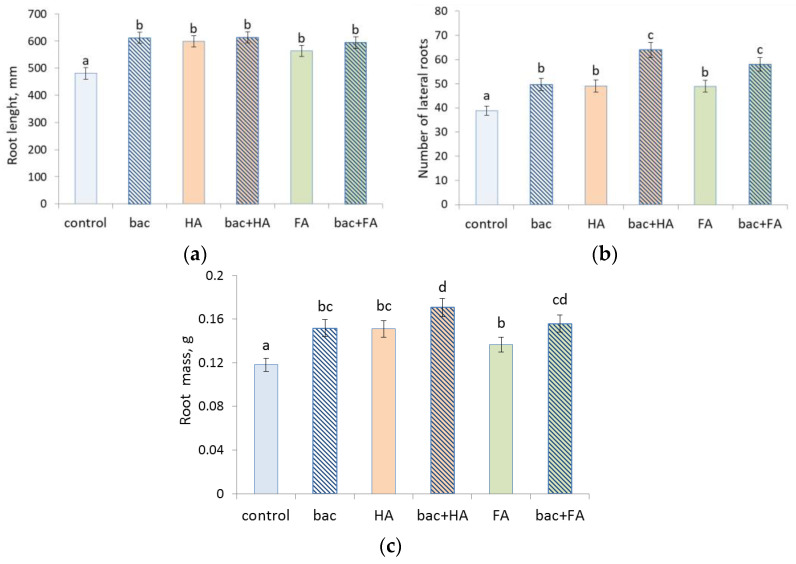
Total length of all seminal roots (**a**), number of lateral roots (**b**), and root mass (**c**) of wheat plants 7 days after treatment with *Pseudomonas plecoglossicida* 2,4-D (bac), humic (HA), and fulvic (FA) acids, and their combinations (bac + HA and bac + FA). Means statistically significant difference from each other are indicated by different letters, *p* ≤ 0.05, *n* = 15 (ANOVA followed by Duncan’s test).

**Figure 2:**
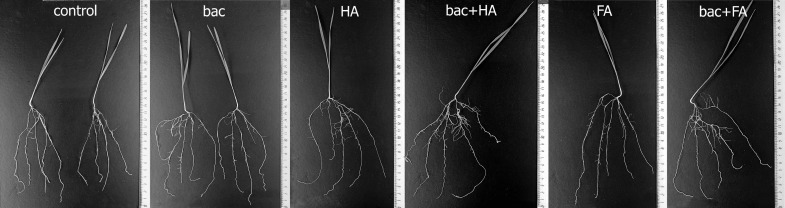
Images of wheat plants 7 days after treatment with *Pseudomonas plecoglossicida* 2,4-D (bac), humic (HA), and fulvic (FA) acids, and their combinations (bac + HA and bac + FA).

All treatment options (the use of HA and FA and bacterial treatment applied separately and in combination) led to an increase in the overall length of seminal roots, their mass, and number of lateral roots of wheat plants compared to the control (untreated plants) (Figure 1 and Figure 2). All treatments resulted in a similar increase in root length, while the impact on the number of lateral roots was greater when HSs were combined with bacteria. HA combined with bacteria led to a greater increase in the number of lateral roots than either bacteria or humic substances (HSs) applied alone. Root mass of plants treated with FA combined with bacteria was significantly heavier than in the case of FA applied alone, and intermediate between plants treated with bacteria + HA and those treated with bacteria and HA applied alone.

**Figure 3:**
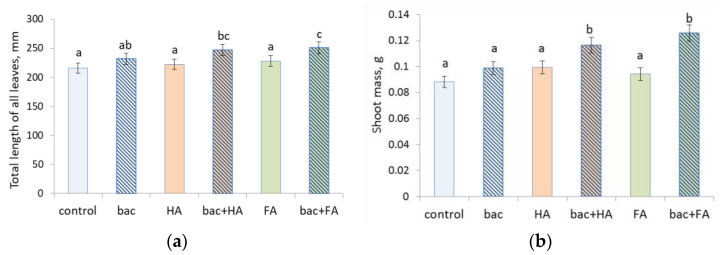
Total length of all leaves (**a**) and shoot mass (**b**) of wheat plants 7 days after treatment with *Pseudomonas plecoglossicida* 2,4-D (bac), humic (HA), and fulvic (FA) acids, and their combinations (bac + HA and bac + FA). Means statistically significant difference from each other are indicated by different letters, *p* ≤ 0.05, *n* = 15 (ANOVA followed by Duncan’s test).

Shoots of wheat plants were significantly longer and heavier than in the control only in plants treated with bacteria combined with HSs (Figure 2 and Figure 3). Nether bacterial treatment nor FA or HA applied alone significantly increased shoot length or mass compared to the control. The tendency of longer and heavier shoots of plants treated with bacteria + FA compared to plants treated with the combination of bacteria and HA was statistically insignificant.

Although a greater increase in the shoot mass of plants treated with bacteria + FA and the opposite effect in roots (greater impact of bacteria + HA than bacteria + FA on the root mass) were statistically insignificant, the difference between them in a root-to-shoot mass ratio was statistically significant (higher in plants treated with bacteria + HA compared to those treated with bacteria + FA: (1.5 ± 0.1) and (1.3 ± 0.1) g/g, respectively, with the difference being significant at *p* = 0.4, *n* = 15.)

**Figure 4:**
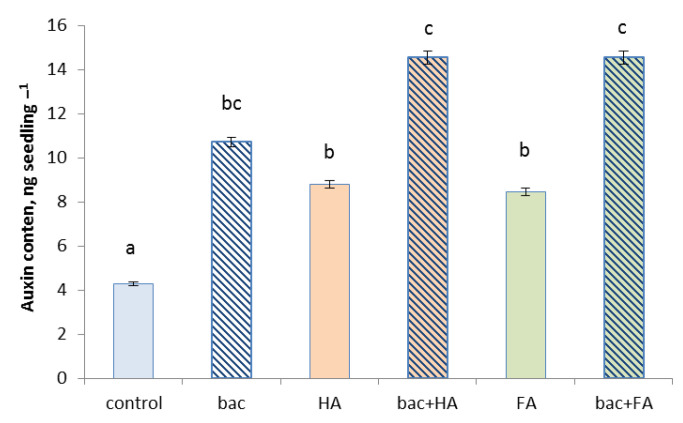
Auxin content in wheat seedling 3 days after treatment with *Pseudomonas plecoglossicida* 2,4-D (bac), humic (HA), and fulvic (FA) acids, and their combinations (bac + HA and bac + FA). Means statistically significant difference from each other are indicated by different letters, *p* ≤ 0.05, *n* = 9 (ANOVA followed by Duncan’s test).

All treatment options (the use of HA and FA and bacterial treatment applied separately and in combination) increased auxin content in wheat seedlings (Figure 4). The greatest effect was detected when the combination of bacteria and HSs was applied.

## 4. Discussion

All the treatments applied in the present research increased root growth parameters (root length and mass and lateral root numbers) compared to the control, which is in accordance with literature data showing growth promotion by either PGPB [1,2,3] or humic substances [4,5,6]. Furthermore, the stimulating effect on root parameters was stronger when plants were treated with a combination of *P. plecoglossicida* 2,4-D with either HA and FA, while in shoots, only these combinations increased shoot mass and length compared to the control, and these parameters did not differ from the control in plants treated with either bacteria or humic substances applied alone. This synergistic effect of combination of humic substances and bacteria has been reported in some articles, which were recently reviewed [15,16]. It was important to investigate whether the increased activity of combined components was due to the action of HA on bacteria or directly on plants.

Addition of HSs to the culture media increased bacterial titer at certain time intervals in vitro, which is in accordance with the reports showing that HA can stimulate the growth of bacteria [17]. In the present experiments, the stimulating effect of humic substances was more clearly manifested in the stationary phase of growth. The main factors initiating the transition of a bacterial culture to the stationary phase of development during artificial cultivation are considered to be nutrient deficiency and the accumulation of toxic metabolic products. It is known that bacterial cells adsorb humic substances on their surface [30], and the adsorption of HA is higher than that of FA [31]. Many authors believe that humic substances can affect the state and permeability of the outer membrane [32], and increase the ability of bacteria to take up nutrients from the environment [33,34]. There is also evidence that humic substances protect microorganisms from the effects of toxic compounds [35,36]. Each of these mechanisms or their combination can be the cause of achieving and maintaining a higher final titer of bacteria in the variants of the experiment with the addition of humic substances. The practical result of the observed phenomenon may be better survival and maintenance of the number of introduced bacteria under a natural environment, since most of the bacteria in the soil are normally in the stationary phase due to limited access to nutrient compounds. This suggestion was confirmed by the results of the estimation of root colonization by bacteria in the present experiments showing that addition of HA increased the number of CFU per g of root mass. These results suggest that the increased stimulating effect of the combination of HA with bacteria is likely to be due to the effect of HA on the growth of bacteria. Still, this is not the only explanation of the synergistic action of the combination of these components, since FA + bacteria was more effective in stimulating plant growth than *P. plecoglossicida* 2,4-D alone, although the effect of FA on bacterial growth in vitro was rather weak. These results suggest that another explanation is possible, which is the effects of humic substances on plants themselves. This suggestion was supported by the data showing promotive effects on plant growth induced by humic substances (FA and HA) applied individually.

All treatment options resulted in an increased number of lateral roots which is likely to be due to the increased concentration of auxins known to be necessary for control root branching [37]. Increased concentration of auxins in plants treated with *P. plecoglossicida* 2,4-D can be related to the ability of this strain to produce auxins and to supply plants with this hormone [38]. HSs have also been shown to increase auxin concentration in plants [6,39]. Bacteria and HSs applied alone or in combination increased auxin content in the wheat seedling in the present experiments (Figure 4), while the effect was the strongest in the case of their combination. Increased root branching is necessary for active uptake of nutrients which promotes plant growth. Thus, the stronger impact of *P. plecoglossicida* 2,4-D combined with either HA or FA is likely to be due to their additive effect on auxin accumulation in the treated plants.

Although both HA and FA increased promotive action of *P. plecoglossicida* 2,4-D on plant growth, the mechanism of their action is unlikely to be identical. The difference between them was best manifested in their action of the root-to-shoot mass ratio in the presence of *P. plecoglossicida* 2,4-D, with HA enabling greater promotion of root growth, while FA was more effective in stimulating shoot growth. Since root growth becomes more important under edaphic stresses while shoot growth enables photosynthesis and productivity under favorable environments, our results can guide the choice of either HA or FA in combination with bacteria as biostimulants used in agriculture depending on the climate.

The greater effect of the combination of HSs with bacteria compared to their separate effects could be due to an increase in bacterial auxin production induced by HS, thereby promoting root development. Although we failed to detect a HS-induced increase in auxin production in vitro, this effect could have taken place when bacteria were associated with plants. Furthermore, both bacteria and HSs increased auxin content in treated plants [6,38,39], which is likely to result in the additive action of their combination on auxin concentration in treated plants. Increased root branching brought about by an elevated auxin concentration provides a greater surface for colonization by bacteria, which is likely to be the cause of the synergistic effect of the HSs–bacteria combination. In the future, longer experiments are needed to confirm the persistence of the effects over time. However, the present short-term experiments were necessary to demonstrate the effects of the treatments on root branching, which would be difficult to detect in older plants.

## Figures and Tables

**Table 1:** Cell number dynamics of bacteria cultured in the King B medium, CFU mL^−1^. Significantly different means in each raw are marked with different letters (*n* = 5, *t*-test, *p* ≤ 0.05).

Time of Incubation, h	Additives to the Medium			
	No Additives (Control)	HA	FA	HSs
0	(3.18 ± 0.14) × 10^4 a^	(3.02 ± 0.21) × 10^4 a^	(3.26 ± 0.23) × 10^4 a^	(2.99 ± 0.17) × 10^4 a^
4	(3.85 ± 0.43) × 10^4 a^	(4.53 ± 0.38) × 10^4 a^	(3.41 ± 0.30) × 10^4 a^	(4.28 ± 0.63) × 10^4 a^
8	(2.27 ± 0.60) × 10^6 a^	(5.57 ± 0.99) × 10^6 b^	(5.37 ± 0.84) × 10^6 b^	(1.80 ± 0.70) × 10^6 a^
24	(1.03 ± 0.14) × 10^9 a^	(1.77 ± 0.29) × 10^9 a^	(1.21 ± 0.09) × 10^9 a^	(1.55 ± 0.20) × 10^9 a^
48	(1.79 ± 0.18) × 10^9 a^	(2.90 ± 0.19) × 10^9 b^	(2.25 ± 0.20) × 10^9 a^	(2.37 ± 0.25) × 10^9 a^
96	(2.02 ± 0.16) × 10^9 a^	(6.35 ± 0.68) × 10^9 c^	(7.09 ± 0.45) × 10^9 c^	(4.19 ± 0.36) × 10^9 b^

**Table 2:** Cell number dynamics of bacteria cultured in Raymond’s media, CFU mL^−1^. Significantly different means in each raw are marked with different letters (*n* = 5, *t*-test, *p* ≤ 0.05).

Time of Incubation, Days	Additives to the Medium			
	No Additives (Control)	HA	FA	HSs
0	(4.07 ± 0.36) × 10^5 a^	(3.85 ± 0.40) × 10^5 a^	(3.80 ± 0.29) × 10^5 a^	(4.11 ± 0.32) × 10^5 a^
1	(4.15 ± 0.53) × 10^6 a^	(1.14 ± 0.30) × 10^7 b^	(2.87 ± 0.42) × 10^6 a^	(7.20 ± 0.68) × 10^6 b^
2	(4.12 ± 0.74) × 10^6 a^	(1.22 ± 0.34) × 10^7 b^	(3.10 ± 0.86) × 10^6 a^	(7.62 ± 0.85) × 10^6 ab^
7	(4.44 ± 0.50) × 10^6 a^	(2.15 ± 0.29) × 10^7 b^	(5.08 ± 0.79) × 10^6 a^	(7.70 ± 0.76) × 10^6 ab^

**Table 3:** Number of colonies formed by bacteria from the root washing in nutrient agar and King B medium with rifampicin, CFU g^−1^ of roots. Minus indicates samples where the number of rifampicin-resistant bacteria was below the detection threshold. Significantly different means in each column are marked with different letters (*n* = 5, *t*-test, *p* ≤ 0.05).

Treatment	Nutrient Medium	
	Nutrient Agar	King B^rif^
**No additives (control)**	(5.48 ± 0.49) × 10^6 a^	-
***Pseudomonas plecoglossicida*** **2,4-D**	(1.88 ± 0.09) × 10^7 d^	(1.70 ± 0.14) × 10^7 a^
**HA**	(9.94 ± 0.55) × 10^6 c^	-
**HA + *P. plecoglossicida*** **2,4-D**	(4.33 ± 0.15) × 10^7 e^	(3.60 ± 0.26) × 10^7 c^
**FA**	(6.86 ± 0.44) × 10^6 b^	-
**FA + *P. plecoglossicida* 2,4-D**	(1.89 ± 0.08) × 10^7 d^	(2.03 ± 0.13) × 10^7 b^

## Data Availability

The data presented in this study are contained within this article.

## Appendix A

**Table A1:** Cell number dynamics of bacteria cultured in Ashby’s media, CFU mL^−1^. Significantly different means in each raw are marked with different letters (*n* = 5, *t*-test, *p* ≤ 0.05).

Time of Incubation, Days	Additives to the Medium			
	No Additives (Control)	HA	FA	HSs
0	(3.91 ± 0.48) × 10^5 a^	(4.15 ± 0.42) × 10^5 a^	(4.03 ± 0.37) × 10^5 a^	(4.17 ± 0.33) × 10^5 a^
1	(1.42 ± 0.22) × 10^7 a^	(3.02 ± 0.51) × 10^7 a^	(1.81 ± 0.28) × 10^7 a^	(2.50 ± 0.40) × 10^7 a^
2	(1.03 ± 0.18) × 10^7 a^	(1.10 ± 0.23) × 10^7 a^	(8.36 ± 0.92) × 10^6 a^	(1.14 ± 0.19) × 10^7 a^
7	(5.81 ± 0.55) × 10^6 a^	(8.05 ± 0.72) × 10^6 a^	(5.07 ± 0.66) × 10^6 a^	(6.10 ± 0.69) × 10^6 a^
